# Gender Recognition from Human-Body Images Using Visible-Light and Thermal Camera Videos Based on a Convolutional Neural Network for Image Feature Extraction

**DOI:** 10.3390/s17030637

**Published:** 2017-03-20

**Authors:** Dat Tien Nguyen, Ki Wan Kim, Hyung Gil Hong, Ja Hyung Koo, Min Cheol Kim, Kang Ryoung Park

**Affiliations:** Division of Electronics and Electrical Engineering, Dongguk University, 30 Pildong-ro 1-gil, Jung-gu, Seoul 100-715, Korea; nguyentiendat@dongguk.edu (D.T.N.); yawara18@dongguk.edu (K.W.K.); hell@dongguk.edu (H.G.H.); koo6190@naver.com (J.H.K.); mincheol9166@naver.com (M.C.K.)

**Keywords:** gender recognition, human body images, convolutional neural network, visible-light and thermal camera videos

## Abstract

Extracting powerful image features plays an important role in computer vision systems. Many methods have previously been proposed to extract image features for various computer vision applications, such as the scale-invariant feature transform (SIFT), speed-up robust feature (SURF), local binary patterns (LBP), histogram of oriented gradients (HOG), and weighted HOG. Recently, the convolutional neural network (CNN) method for image feature extraction and classification in computer vision has been used in various applications. In this research, we propose a new gender recognition method for recognizing males and females in observation scenes of surveillance systems based on feature extraction from visible-light and thermal camera videos through CNN. Experimental results confirm the superiority of our proposed method over state-of-the-art recognition methods for the gender recognition problem using human body images.

## 1. Introduction

Recently, surveillance systems have become highly popular and have many useful applications. A common application of surveillance systems is remote video monitoring in private houses, businesses, or outdoor environments [1,2,3] to monitor the premises and/or to prevent crime, as well as for monitoring the people who enter or leave. This type of information is important for security purposes. In some public areas, such as parks, schools, roads, parking lots, and places for celebrating events, such as stadiums and music concert venues, surveillance systems can be used to monitor for criminals and/or malicious human actions, road traffic conditions, and other purposes. Surveillance systems enable users to either manually or automatically manage the observation scene, and to detect criminal activity sooner. To accomplish these functions, the surveillance system is required to capture successive images and extract useful information from the scene under observation, such as the appearance of people (entering or leaving the observation scene) [1], their gender [4,5], and age and/or actions [1]. The gender information of a person is an important feature in surveillance systems [4,5]. In businesses, shop owners can show different advertisements of their products to customers standing in front of an advertising board according to their gender (male and female). This scheme offers a dynamic and useful advertisement strategy. The surveillance systems can also be used to monitor customers and collect demographic information. For example, a shop owner can use a surveillance system to obtain information on how customers of different gender and age groups interact with specific goods. From this information, the shop owner can capture shopping trends and build a business plan. The gender information can also help security systems to limit criminal activity and control access of males or females to restricted areas. Therefore, gender recognition is an important task in surveillance systems.

Most of the previous studies on gender recognition methods used facial images that offer very high recognition accuracy [6,7,8,9]. However, the use of facial images is not suitable for gender recognition in surveillance systems because of the distance between the observed person and the camera. In addition, the use of facial images for gender recognition requires the cooperation of the person, which is normally not available in surveillance systems. Consequently, the use of human body images can be considered an alternative method of gender recognition in surveillance systems. However, only a few studies have addressed the recognition of gender information in a surveillance system using images of the human body. A study conducted by Cao et al. [10] proved that it is possible to recognize the gender of a person using visible-light images of the human body only. In this study, they applied the predesigned feature extraction method, i.e., the histogram of oriented gradients (HOG) feature, on visible-light images of the human body and used a boosting method to recognize the gender of the person. The experimental results on a public database (called the MIT database) that contains ~1000 images showed that they could obtain a recognition accuracy of ~75%. To enhance recognition accuracy, Guo et al. [11] used another predesigned feature extraction method, called biologically inspired features (BIFs), and a support vector machine (SVM) on the same database. Using these methods, they reported that the correct recognition accuracy was enhanced to ~80%, higher than recognition accuracy of the previous research by Cao et al. [10]. In these studies, the authors used only visible-light images for gender recognition. Therefore, the recognition accuracy is limited because of the large variation of the human body in visible-light images, such as clothing, accessories, background, and other factors.

To overcome this problem, Nguyen et al. [5] used additional gender information sourced from thermal images of the human body for gender recognition. Given that thermal images are captured based on the difference in temperature between the human body and background regions, the thermal images are less affected by the variation of clothing or accessories. Consequently, the thermal images are more efficient in describing the human body shape than the details on the human body. Using two types of images, Nguyen et al. [5] also applied the HOG feature extraction method for feature extraction and SVM for gender recognition. Their experimental results showed that the recognition accuracy of the combined images was better than that of using only visible-light or only thermal images for gender recognition. They also used a special characteristic of thermal images, where background regions appear darker than the human body regions despite their complex structure, to assess the quality of sub-regions in the visible and thermal images to construct a new feature called weighted HOG [12]. This method is efficient for reducing the effects of background regions on the extracted HOG features and consequently helps to enhance the recognition accuracy. In addition to the use of a single image for gender recognition, several other studies have used a sequence of images [13,14] or the three-dimensional (3D) shape of the human body [15,16] for gender recognition. Although the methods mentioned previously have demonstrated recognition of gender information from images of the human body, their recognition accuracy is limited by the use of predesigned and/or unsuitable feature extraction methods, such as HOG, BIFs, or weighted HOG. The use of an unsuitable feature extractor can result in unsuitable extracted features and associated noise, which reduces recognition accuracy. To overcome the limitations of the predesigned feature extractors, we propose a new gender recognition method that uses a more suitable feature extractor based on a convolutional neural network (CNN). Our study is novel in the following four aspects compared to previous methods:

- First, we collected a database of visible-light and thermal images of the human body for gender recognition purposes. To the best of our knowledge, there is little previous research on body-based gender recognition using both visible-light and thermal images. As a result, there is no public database for evaluating the performance of such gender recognition systems. Therefore, we make this database available for other researchers to use in their work, from which development and comparison can be performed.
- Second, we designed and trained CNN models for gender recognition using human body images from visible-light and thermal cameras to build the most suitable feature extractor for each type of image.
- Third, we extract the features from visible-light and thermal images using the trained CNN models. Given that the CNN model is trained using a large number of human body images with respect to the gender information, it is a more suitable feature extractor for the body-based gender recognition problem with our database than other feature extraction methods, such as the HOG, BIFs, or weighted HOG.
- Fourth, we combine the extracted image features (by the CNN method) from the visible-light and thermal images and perform noise and feature dimension reduction by principal component analysis (PCA). Finally, gender classification is performed by SVMs to enhance the recognition accuracy.

The fusion of visible-light and IR bands has been used to improve the performance of some biometrics systems such as multispectral face-based human identification and palm-print with palm-vein recognition. However, to the best of our knowledge, previous studies on gender recognition systems have mostly used only the face or visible-light human body images [6,7,8,9,10,11]. To enhance the performance of these approaches, previous studies addressed body-based gender recognition using two types of images (visible-light and thermal images) [5,12]. However, they have the disadvantage that the feature extractors are manually designed, by which the enhancement of accuracy is limited with the images captured in various environments. Therefore, we enhance the performance of body-based gender recognition by combining visible-light and thermal images with CNN, from which high-performance feature extractors can be automatically obtained by an intensive learning procedure with an extremely large amount of data captured in various environments. A comparison of previous studies on body-image-based gender recognition with our proposed method is summarized in Table 1.

The remaining sections of this paper are organized as follows: in Section 2, we describe the overall procedure of our proposed gender recognition method using CNN for image feature extraction and SVM for gender recognition. In Section 3, we present the recognition accuracy of our proposed method in comparison with previous methods by performing various experiments to demonstrate the efficiency of our proposed method. Finally, we present the concluding remarks in Section 4.

## 2. Proposed Method

### 2.1. Overview of the Proposed Method

**Figure 1:**
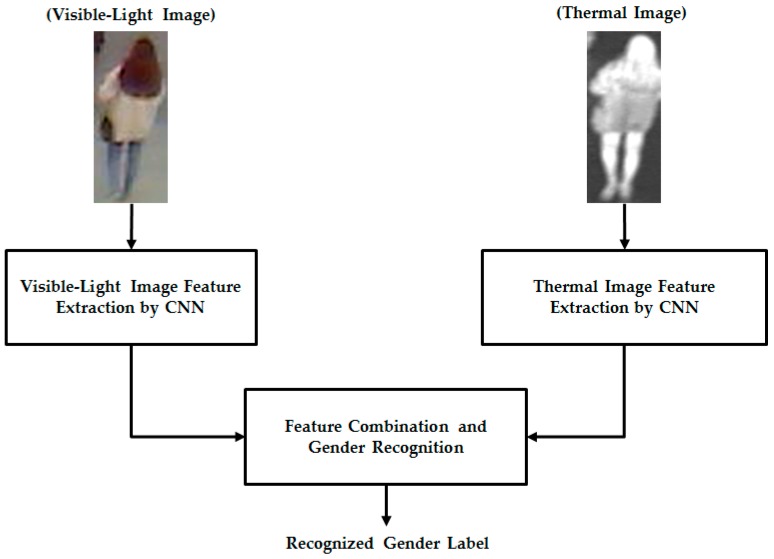
Overview of our proposed method for gender recognition using CNN for image feature extraction.

In this study, we used two different types of images of the human body for the gender recognition problem. As mentioned in Section 1, the previous studies by Cao et al. [10] and Guo et al. [11] used only visible images for the gender recognition problem. Owing to the large variation of the human body, such as the variation of clothes, accessories, hair styles, and other factors, gender recognition accuracy using only single visible-light images is limited. Therefore, as suggested by Nguyen et al. [5,12], this study used an additional type of human body image, called a thermal image, to enhance the recognition accuracy. The details of our proposed method are presented in Figure 1.

In the proposed gender recognition method, the human body images are first captured using two cameras, namely a visible-light camera and a thermal camera, to obtain the corresponding visible-light images and thermal images. For the preprocessing step, we use a human detection method to localize the human body regions in the captured images. In this study, we focus only on gender recognition; therefore, we use a human detection method by Lee at al. [17] for this step. As a result, we can obtain the localized human body images as shown in Figure 1 (“Visible-Light Image” and “Thermal Image”). With these two input images, our proposed method uses two trained CNN models to extract the image features for visible-light and thermal images, instead of using the predesigned feature extraction methods, such as HOG, BIFs, or weighted HOG used in previous studies [5,10,11,12]. CNN is a learning-based method for image classification and has been used in various applications. Our method trains the CNN model using a large number of human body images with respect to gender information. The principle of the CNN method and the details of the CNN architecture used in this study are presented in Section 2.2.

Finally, the extracted features from the visible-light and thermal images using the CNN method are combined and the gender is recognized using SVM. As suggested by Nguyen et al. [5,12], our proposed method combines visible-light and thermal images using two combination methods, i.e., feature-level fusion and score-level fusion, for gender recognition. Detailed explanations of the feature extraction and gender recognition are presented in Section 2.3.

### 2.2. CNN-Based Feature Extraction Method for Gender Recognition

**Figure 2:**
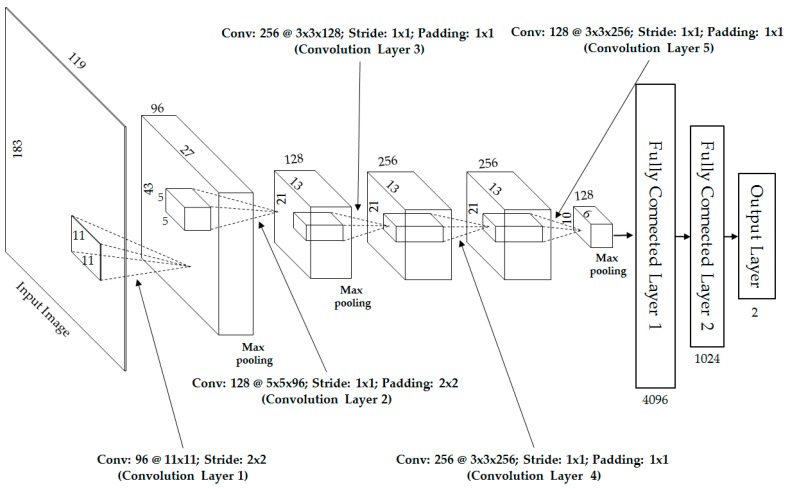
Design architecture of our CNN for gender recognition using visible-light or thermal images.

Previous studies on human body-based gender recognition are mainly based on predesigned (hand-designed) feature extractors, such as local binary patterns (LBP), HOG, BIFs, and weighted HOG, and followed by classification methods. The limitation of this approach is that the same feature extractor is used at all locations in the images despite texture differences between locations. In addition, the design of the feature extractors is based on the observation and knowledge of the designers on a specific problem. Therefore, the extractors simply capture several aspects of the problem. For example, the LBP method is designed to count the number of uniform and non-uniform image texture features in an image [5,18], the HOG method is designed to capture the edges and edge strengths [5,10,12,19], and the BIFs method is designed to extract the image features using different bandwidth and texture direction using Gabor filters [11]. Given the predesigning approach, these image feature extractors have fixed parameters and definitions even though they are applied to various types of images and/or different image textures. Consequently, the extracted image features are weak and the consequent recognition results are limited. To overcome this problem, we propose the use of CNN, which is a learning-based method, for image feature extraction instead of using the predesigned methods. The overall architecture of our CNN comprising five convolutional layers and three fully connected layers is shown in Figure 2. In addition, the detailed descriptions of the network are given in Table 2 to explain the details of Figure 2. In Table 2, “n/a” stands for “not available.”

The main structure of a CNN is convolutional layers followed by the rectified linear units (ReLUs) and pooling layers [20,21,22,23,24,25,26,27,28,29,30]. As reported in previous studies, the CNN method has been successfully applied for many computer vision systems and produced superior results compared to traditional methods. For example, the CNN method has been successfully used for handwriting recognition [20], image classification [21], face recognition [22], image-depth estimation from a single color image [23], person re-identification [24,25], facial trait recognition [26], gaze estimation [27], lane estimation [28], eye tracking [29], and face detection [30]. As shown in Figure 1, the inputs of the CNN network are the detection results of human body regions from visible-light and thermal images. For the processing step, we performed a size normalization step and made all human-body-region images to be the same size of 183 × 119 pixels (height × width). The size normalization step is used to compensate the difference between the near and far capture images and to align the input images. In addition, we also normalize the image’s illumination by performing the zero-center method [31].

For the first convolutional layer of our CNN structure, the input images of 183 × 119 pixels (visible-light or thermal images) are given to a convolutional layer with 96 filters of size 11 × 11 pixels at a stride of 2 × 2 pixels in the horizontal and vertical directions. To make the CNN structure robust to image translation, the 96 feature maps are fed to a max-pooling layer. As a result, the outputs of the first layer are 96 feature maps of size 43 × 27 pixels, as shown in Figure 2 and Table 2. To fine-tune the first layer, we placed the second layer after the first layer with 128 filters of size 5 × 5 × 96, a stride of 1 pixel, and padding of 2 pixels, followed by another max-pooling layer. Using the first two layers, we obtain 128 feature maps of size 21 × 13 pixels, as shown in Figure 2 and Table 2. The first two layers are used to extract the low-level image features, such as edges or blob texture features.

For the high-level feature extraction, we use three additional convolution layers as shown in Figure 2 and Table 2. In detail, the third layer has 256 filters of size 3 × 3 × 128, the fourth layer has 256 filters of size 3 × 3 × 256, and the fifth layer has 128 filters of size 3 × 3 × 256. Using these five convolutional layers, we obtain 128 feature maps of size 10 × 6 pixels. These feature maps are fed to the three fully connected layers that include 4096, 1024, and 2 neurons, respectively. Given that we are addressing the gender recognition problem, there are only two possible output values of the CNN architecture, namely, male and female. Therefore, the last fully connected layer (called the “Output Layer” in Figure 2) contains only two neurons. To extract the image feature, we use the features at the second fully connected layer. Consequently, we can extract a feature vector of 1024 components (a vector in 1024-dimensional space) for each visible-light or thermal image.

As reported in previous studies [21,32], CNN-based systems are usually faced with the over-fitting problem. This problem can cause poor recognition accuracy of the testing phase although the accuracy of the training phase is still good. To reduce the over-fitting problem, our proposed method uses two methods, namely, the data augmentation and dropout methods [21,32], which were demonstrated to help reduce the effects of the over-fitting problem in CNN networks. For the first method, we artificially make augmented data from the original data by removing some pixels on the left, right, top, and bottom sides of the original images [21]. For the second method, we apply a dropout value (probability value) to disconnect the connections of several neurons between a previous layer and the next layers in the network [21,32].

Unlike the AlexNet [21], we designed a simpler CNN architecture using five convolution layers and three fully connected layers. The AlexNet and some other studies [20,22] normally used the input images in a square shape. In order to use this scheme, the images of objects are scaled to a square size before feeding to the CNN network. However, in our research on gender recognition using human body images, the natural height of the body images is approximately double the width. Therefore, in our design, the size of the input images is only 183 × 119 pixels (height-by-width), much smaller than the 227 × 227 × 3 size in the AlexNet [21]. Using this image size, we tend to reduce the problem of image distortion caused by incorrect human body region detection and image scaling. In addition, the number of filters in each convolution layer and the number of neurons in fully connected layers are also much smaller than those in the AlexNet. Originally, the AlexNet was designed for classifying images into 1000 classes [21]. As a result, it requires a more complex structure. In our research, there are only two classes, male and female. Therefore, this design can help us to reduce the complexity of the network, reduce the training time, and obtain the better classification results compared to conventional methods such as HOG and weighted HOG.

### 2.3. Feature Combination and Gender Recognition Using SVM

As shown in Figure 1, our proposed method uses the trained CNN models that were obtained using visible-light and thermal images separately through a training process to extract the image features for the gender recognition problem. To extract the image features, the input image is first transmitted through the CNN network, and the extracted features are obtained from the second fully connected layers in Figure 2. Consequently, we obtain a feature vector of 1024 components for each input visible-light or thermal image. As suggested in previous studies [5,12], the extracted image features from two types of images can be combined using two combination methods, namely, feature-level fusion and score-level fusion. In this study, we performed both combination methods using a new type of image feature (CNN-based feature), and compared the performance of this method with previous conventional methods, such as HOG and weighted HOG.

**Figure 3:**
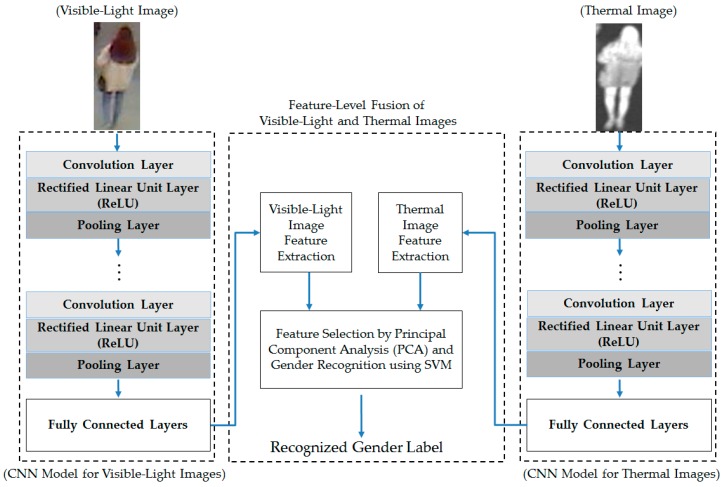
Feature-level fusion combination method for gender recognition using visible-light and thermal images of the human body.

For the feature-level fusion combination method, the extracted image features from the visible-light and thermal images using the CNN method are concatenated together to form a new combined feature, called the fusion feature, as shown in Equation (1) [5,12]. In this equation, *f_v_* and *f_t_* are the image features extracted from the visible and thermal images. The concatenation of the image features of the two types of images results in the fusion feature *f_c_*. A detailed flow chart of this combination method is shown in Figure 3.

(1) $$f_{c}=\left[f_{v\;},\;f_{t}\right]$$

**Figure 4:**
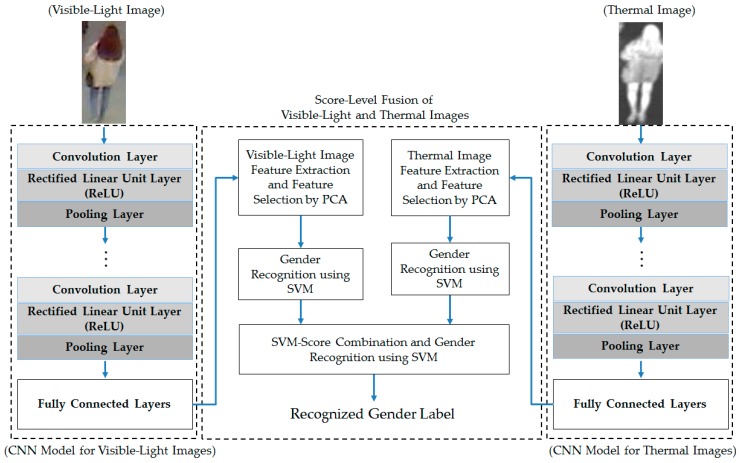
Score-level fusion combination method for gender recognition using visible-light and thermal images of the human body.

For the second combination method, score-level fusion, gender recognition by single visible-light and thermal images is first performed using the SVM method. Consequently, we obtain two scores (decision values) that stand for the probabilities of the input visible-light and thermal images belonging to the male and female classes. These scores are then used as two inputs to another SVM; from the output of this SVM, the final classification of gender can be made. Figure 4 shows the methodology of this combination method.

As shown in Figure 3 and Figure 4, our proposed method uses the SVM to recognize the gender labels (male and female) of the input images. The SVM is a well-known supervised learning method in machine learning and is mainly used for classification and regression problems using support vectors. With training data, the SVM method will learn to obtain several support vectors, *x_i_*, as well as classifier parameters. Finally, the class label of a test image is evaluated using Equation (2). In this equation, *y_i_* is the class label corresponding to the support vector *x_i_*; *K*(·) is the kernel function that is used to map the input data from low-dimensional to higher-dimensional feature space. In our experiments, we use two different SVM kernels, including the linear and radial basis function (RBF) kernel, as shown in Equations (3) and (4). In addition, we use the open-source LibSVM library for the SVM implementation [33]:

(2) $$f\left(x\right)=sign(\overset{k}{\underset{i=1}{\sum }}a_{i}y_{i}K\left(x,x_{i}\right)+b)$$

(3) $$\mathrm{Linear}\;\mathrm{kernel}:\;K\left(x_{i},x_{j}\right)={x_{i}}^{T}x_{j}$$

(4) $$\mathrm{RBF}\;\mathrm{kernel}:\;K\left(x_{i},x_{j}\right)=e^{-\gamma \|x_{i}-x_{j}{\|}^{2}}$$

As shown in previous studies [5,10,11,12], human body images have very large variation caused by differences in background, clothing, accessories, body poses, hair styles, and other factors. Therefore, although we use a CNN-based method that can be seen as a suitable feature extractor for image feature extraction, the extracted features can contain little redundant information. As suggested by previous studies [5,11,12,34,35], our proposed method uses principal component analysis (PCA) to reduce the noise and feature dimension before recognizing the gender. The PCA is a well-known method that uses algebra to reduce the dimension of data by finding a new coordinate system in a low-dimensional data space to represent the original data. Suppose that we extract a feature vector *f_i_* in *n*-dimensional space for an input image *x_i_* of a training database. The PCA method will construct a transformation matrix *W* for a new lower *m*-dimensional space (*m* is smaller than *n*) using *m* eigen-vectors that correspond to the *m* largest eigen-values of the covariance matrix C in Equation (5). In this equation, *N* indicates the number of feature vectors in the training database, $\bar{f}$ indicates the mean feature vector, and *T* is the transpose operator. Finally, a new input feature *f* in *n*-dimensional data space can be projected to the new *m*-dimensional space using Equation (6) to obtain the new representation of the feature in low dimensional space:

(5) $$C=\frac{1}{N}\overset{N}{\underset{i=1}{\sum }}(f_{i}-\bar{f}){\left(f_{i}-\bar{f}\right)}^{T}$$

(6) $$y=W^{T}f$$

## 3. Experimental Results

### 3.1. Experimental Database and Experimental Setups

**Figure 5:**
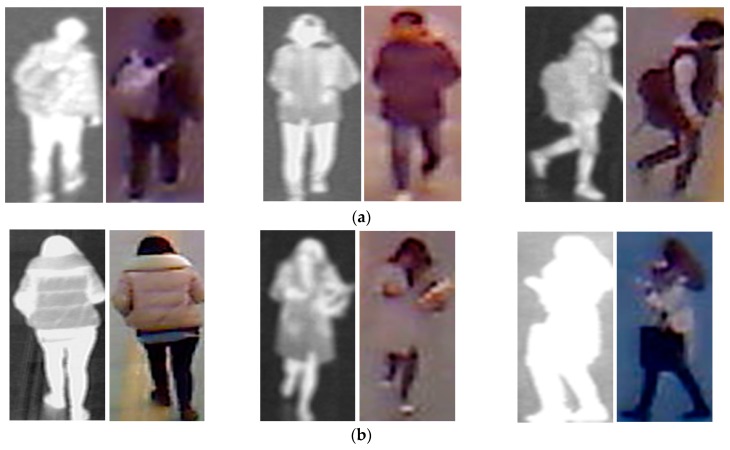
Sample images in our self-established collected database: (**a**) thermal-visible image pairs of male persons; and (**b**) thermal-visible image pairs of female persons.

Given that our proposed method uses two different types of human body images for gender recognition, i.e., visible-light and thermal images, as shown in Figure 1, it is necessary to obtain a pair of visible-thermal images simultaneously to recognize the gender of the observed human in our proposed method for gender recognition. Although there are several open databases of the human body images, such as a visible-light database [36,37,38,39,40] or thermal database [41], these databases cannot be used by our proposed method because they contain only single type of human body image (only visible-light or only thermal images). To the best of our knowledge, a public dataset does exist that contains visible-light and thermal images of pedestrians simultaneously that is dedicated to the pedestrian detection problem [42]. However, this dataset was captured at a very far distance between the people and the camera. In addition, the number of persons is too small to be used for gender recognition problem (approximately 40 persons). Therefore, to measure the recognition accuracy of our proposed method, we used our self-established database [12]. A detailed description of our database is given in Table 3. The database contains images from 412 male and female people comprising 254 males and 158 females. For each person, we captured 10 visible-light images and 10 corresponding thermal images. Consequently, we captured 8240 images (4120 visible-light images and 4120 thermal images). All images were captured using our lab-made dual visible-light and thermal cameras that were placed at a height of approximately 6 m above the observation scene to simulate the actual operation of surveillance systems [12]. To the best of our knowledge, there is little previous research on body-based gender recognition using both visible-light and thermal images. As a result, there is no public database for evaluating the performance of such gender recognition systems. Therefore, we make this database available for other researchers to use in their work, from which development and comparison can be performed [43]. Some sample human-body-region images in our database (human body region images) are shown in Figure 5. As shown in this figure, our database contains male and female images with a large variation of texture and body poses.

To measure the accuracy of the recognition system, we performed a five-fold cross-validation method. For this purpose, we iterate a division procedure that divides the entire database in Table 3 into learning and testing sub-databases five times. In each division, we use images of approximately 80% of the number of males and females to form the learning sub-database, and the other images of the remaining number of males and females are assigned to the testing sub-database. As a result, we obtain five learning sub-databases and five testing sub-databases. Each learning sub-database contains images of 204 males and 127 females, whereas each testing sub-database contains images of 50 males and 31 females.

As discussed in Section 2.2, to reduce the over-fitting problem, we manually made the augmentation database from the original database to enlarge the size of the database [21]. In addition, given that the number of males is larger than the number of females in our database (254 males versus 158 females in Table 3), we intend to make the number of augmented images for males smaller than the number of augmented images for females to make the number of images of males and females similar. For this purpose, we made 18 images from each male image and 30 images from each female image by removing two or four pixels from the left, right, top, and bottom sides of the original image. Consequently, we obtained an augmented database for each learning and testing sub-database as shown in Table 4, where a detailed description of each learning and testing sub-database is shown.

Similar to previous studies on body-based gender recognition [5,12], we used the equal error rate (EER) to evaluate the performance of the gender recognition system. The EER is a principal error measurement that has been widely used in recognition systems, such as finger-vein recognition [44,45], iris recognition [46,47], and face recognition [48]. By definition, the EER is the error when the false acceptance rate (FAR) is equal to the false rejection rate (FRR). In our case of gender recognition, we have two classes, male and female. Therefore, we have two possible error cases of “a ground-truth male image that is falsely recognized as female image” and “a ground-truth female image that is falsely recognized as a male image”. In this study, we call the first case of error, where a ground-truth male image is falsely recognized as a female image, as the FAR; and the other error is the FRR. In biometrics studies, we normally use the genuine acceptance rate (GAR) instead of the FRR value for EER calculation. The GAR is defined as (100 − FRR) (%). With the recognition system, we always hope that the error is as small as possible. Therefore, a smaller value of EER indicates a better recognition system. In our experiments, the final recognition accuracy (EER) of the recognition system is measured by averaging the EERs of five testing databases. In addition, the FAR-GAR pair-value is shown in bold type at the corresponding EER point in our experimental results in Table 5, Table 6, Table 7, Table 8 and Table 9.

### 3.2. Gender Recognition Accuracy Assessment

**Figure 6:**
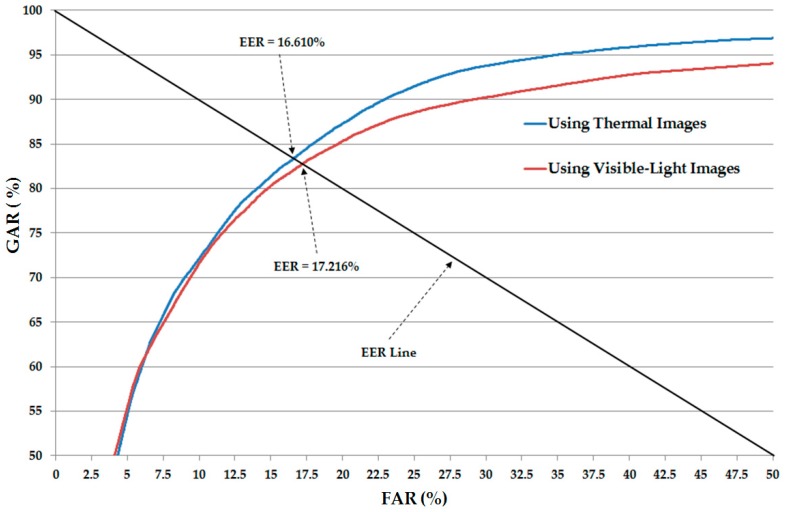
Average ROC curves of recognition systems using single image types with the CNN-based method in Figure 2.

In our first experiment, we performed the training/testing procedures to train the CNN-based feature extractor model for visible-light images and thermal images, respectively, using the CNN structure (in Figure 2) and only single visible-light and only single thermal images. In Table 4, we describe the training and testing databases, which contain both visible-light and thermal images. Therefore, in this experiment, we used 74,820 images (36,720 male images (204 × 10 × 18) and 38,100 female images (127 × 10 × 30)) as training data and 18,300 images (9000 male images (50 × 10 × 18) and 9300 female images (31 × 10 × 30)) as testing data for recognition systems that use only visible-light images and thermal images for gender recognition, respectively. For training the CNN model, we used the MATLAB implementation [31]. In addition, we set the number of epochs to 60. The initial learning rate is 0.01 with a learn-rate-drop factor of 0.1 after every 20 epochs. The detailed experimental results are shown in Table 5. In addition, we show the receiver-operating curve (ROC) of the CNN-based recognition system using only single visible-light and only thermal images in Figure 6. As shown in Table 5 and Figure 6, the average EER of the CNN-based recognition system using only visible-light images is approximately 17.216%, and the average EER of the CNN-based recognition system using only thermal images is approximately 16.610%. As shown in Table 10, these recognition results are comparable to the recognition results of recognition systems that use only visible-light or only thermal images for the gender recognition problem, as shown in previous studies [5,10,11,12]. However, the recognition result using visible-light images is slightly worse than that of the system using the weighted HOG feature extraction method. The reason is that the weighted HOG method estimates the effects of background regions that could have strong effects on the extracted image features using visible-light images. As shown in Figure 5, the background regions of thermal images are much darker than the foreground regions (human body regions). Therefore, the recognition result of a system that uses the CNN-based method and thermal images is better than that of systems using HOG or weighted HOG in previous studies [5,12].

Using the CNN-based method, we can perform gender recognition using either only single visible-light images or only thermal images. Therefore, to combine the visible-light images and thermal images for gender recognition problem, our proposed method uses the CNN-based model for image feature extraction and the SVM for classification. In the next experiment, we performed gender recognition using only visible-light images or only thermal images on the basis of feature extraction by the CNN-based method and classification using SVM. For this purpose, the pre-trained CNN-based models obtained from the first experiment were saved and used to extract the image features of all images in our database shown in Table 4. With the extracted image features, we performed the gender recognition on the basis of SVM [5,12]. As discussed in Section 2.3, our proposed method intends to use PCA for noise and feature dimension reduction. To demonstrate the efficiency of the PCA method on the recognition system, we measured the recognition accuracies in both cases of with and without PCA for comparison purposes. In addition, two types of SVM kernels viz. linear and RBF kernels are used to classify the gender using SVM. The detailed experimental results of this experiment are shown in Table 6 and Table 7. In Table 6, we show the recognition accuracies of the recognition system that uses only visible-light images or only thermal images without applying PCA on the extracted image features. As shown in this table, the recognition accuracies (EER) using the linear kernel are 17.379% and 16.560% using visible and thermal images, respectively. Using the RBF kernel, the recognition accuracies are 17.379% and 16.510%. These experimental results are quite similar to those of Table 5. Therefore, we find that the recognition performance produced by the SVM method is similar to that of the CNN-based method.

**Figure 7:**
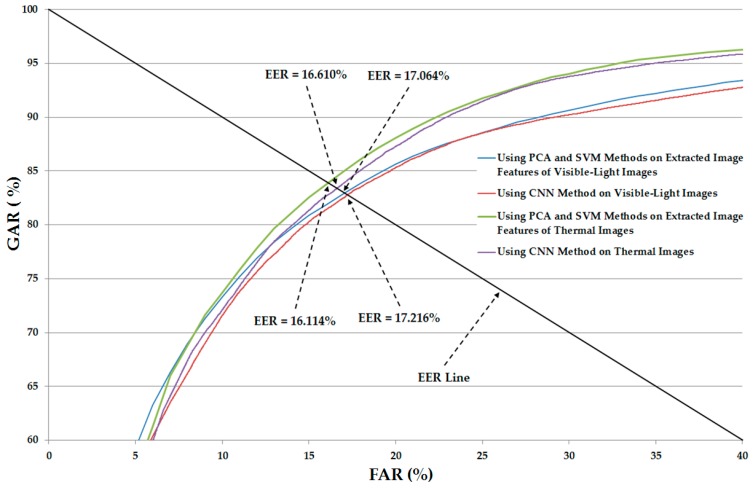
Average ROC curves of the recognition systems using single image types with the CNN-based method and SVM-based method.

Table 7 shows the recognition accuracies of the recognition system, and is similar to Table 6 except for the case of applying the PCA on the extracted image features for noise and feature dimension reduction. As shown in this table, the linear kernel outperforms the RBF kernel for gender recognition. In detail, using only visible-light images, we obtained an EER of 17.064%, which is lower than the EER of 17.489% produced by RBF kernel. This recognition accuracy is also lower than that of the recognition system that uses the CNN-based method (EER of 17.216% in Table 5) and the system that uses SVM without PCA (EER of 17.379% in Table 6). Using only thermal images, we obtained an EER of 16.144%. This EER result is also lower than the EER of 16.610% of the system that uses the CNN-based method (Table 5) and 16.510% of the system that uses the SVM without PCA (Table 6). From the experimental results in Table 5, Table 6 and Table 7, we conclude that gender recognition based on the SVM method is comparable with that of the CNN-based method when using a single type of human body image for gender recognition. In addition, the PCA method is sufficient for enhancing the recognition accuracy. In Figure 7, we show the ROC curves of various recognition system configurations that use only visible-light or only thermal images for gender recognition. This figure again confirms that the recognition system that uses the SVM and PCA methods (SVM-based method) for gender recognition outperforms the recognition system that uses the CNN-based method.

From these experimental results, we performed our next experiment to combine the visible-light and thermal images of the human body for the gender recognition problem. As discussed in Section 2.3, we used two approaches for combining the visible and thermal images, including feature-level fusion (as detailed in Section 2.3 and Figure 3) and score-level fusion (as detailed in Section 2.3 and Figure 4). In addition, we again performed the experiment for two cases, with and without applying PCA for noise and feature dimension reduction, to confirm the efficiency of the PCA method for the gender recognition problem. The experimental results are shown in Table 8 and Table 9 for the cases with and without PCA, respectively.

In Table 8, we show the recognition accuracies of the recognition system that uses the combination of visible-light and thermal images without applying the PCA method. As shown in Table 8, the best recognition accuracy using feature-level fusion was obtained with an EER of 11.684% using the RBF kernel. Using score-level fusion, the best EER is 11.850% using the RBF kernel in the first SVM layer and the linear kernel in the second SVM layer. Compared to the recognition accuracies produced by systems that use only visible or only thermal images for recognition in Table 5 and Table 7 (EER of 17.064% using visible images and 16.114% using thermal images), we can conclude that the combination of visible and thermal images is much more efficient for enhancing the gender recognition problem. As shown in Table 10, this recognition accuracy is also much better than those using the HOG, entropy-weighted histograms of oriented gradients (EWHOG), or weighted HOG methods in previous studies [5,12].

Similar to Table 8, Table 9 shows the recognition results except for the case of using PCA for noise and feature dimension reduction. By applying the PCA method on the CNN-based features, we reduce the recognition error (EER) from 11.684% (in Table 8) to 11.439% using the feature-level fusion approach and linear kernel. Using the score-level fusion approach, we reduce the recognition error from 11.850% to 11.713% using the RBF kernel in the first SVM layer and a linear kernel in the second SVM layer. This result again confirms that the PCA method is sufficient for enhancing the recognition performance. This result also shows that the linear kernel is more sufficient than the RBF kernel for gender recognition using CNN-based feature extraction method.

**Figure 8:**
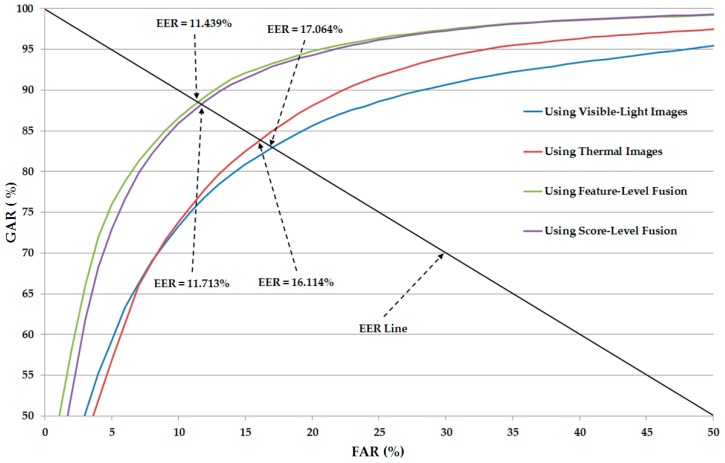
Average ROC curves of recognition systems using our proposed method.

Figure 8 shows the average ROC curves of various system configurations, including the systems using only visible-light or only thermal images for gender recognition and the systems using the combination of visible-light and thermal images (feature-level fusion and score-level fusion methods) for gender recognition. As shown in this figure, the systems using the combination of visible-light and thermal images produced the higher recognition accuracies compared to the systems using a single type of human body images (only visible-light or only thermal images) for the recognition problem. This figure again confirms that the combination of two types of human body images can help to enhance the recognition accuracy.

In Table 10, we summarize the recognition accuracies of our proposed method using single visible-light images, using single thermal images, and the combination of the two types of images in comparison with previous studies. As shown in this table, the best recognition accuracy (EER of 11.439%) was obtained using our proposed method with feature-level fusion. This result is much better than the other recognition results using previous methods in [5,12] and the use of only a single type of images (only visible-light or only thermal images). From this result, we find that our proposed method outperforms the previous studies for the human-body-based gender recognition problem.

**Figure 9:**
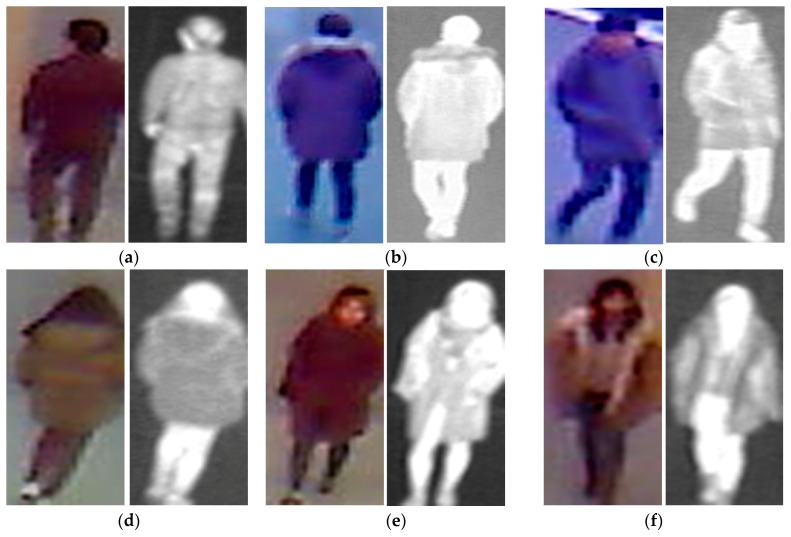
Examples of correct recognition results using our proposed method: (**a**–**c**) examples of male images correctly recognized as males, and (**d**–**f**) examples of female images correctly recognized as females.

**Figure 10:**
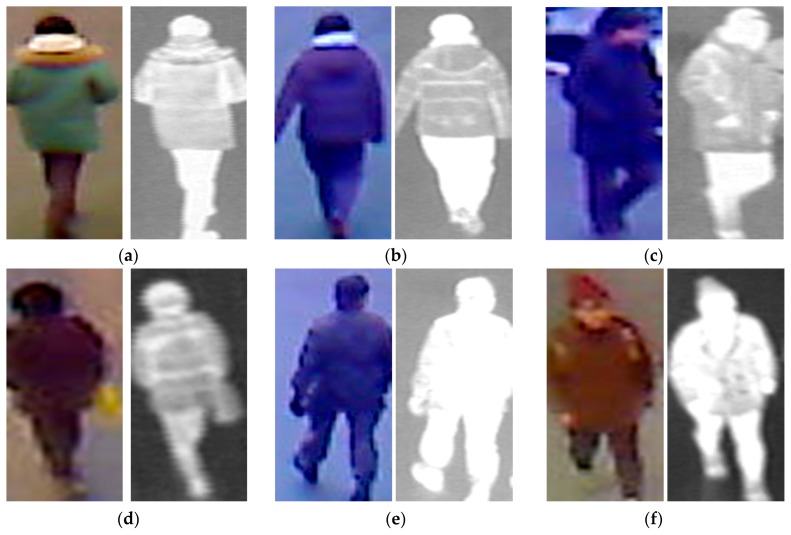
Examples of errors using our proposed method: (**a**–**c**) examples of male images incorrectly recognized as females, and (**d**–**f**) examples of female images incorrectly recognized as males.

For demonstration purposes, we show some sample recognition results by our proposed method in Figure 9 and Figure 10. As shown in Figure 9, our proposed method successfully recognized the gender of people in images regardless of front or back view. However, Figure 10 shows some recognition error cases produced by our proposed method. Figure 10a–c shows error cases when the ground-truth male images were incorrectly recognized as female images. Figure 10d,f shows error cases when the ground-truth female images were incorrectly recognized as male images. From this figure, the appearance of the human body in these images is ambiguous. The images in Figure 10a,b were captured from the back view when the people wore winter clothes. As a result, it is difficult to recognize their gender (male) even by human perception. In Figure 10c, the recognition failed because of the complex background regions. A similar situation also occurred in Figure 10d–f. In Figure 10d, the images of a female person were captured from the back view. In addition, the persons in both cases (Figure 10d,f) wore winter clothes. The female person in Figure 10e wore a military uniform. As we can see from this figure, wearing winter and/or uniform clothes can reduce the distinction between male and female. It is difficult for us to recognize the gender in these sample images despite using human perception. In addition, the surveillance system captures images at a distance from the camera (approximately 10 m). Therefore, the quality of the captured images is normally not good. These negative effects cause the consequent errors of the recognition system.

As shown in our experimental results (Figure 8 and Table 8 and Table 9), the linear kernel outperformed the RBF kernel by producing a lower error (EER) value. In addition, the recognition accuracy of systems with PCA is better than those of systems without PCA (Table 8 and Table 9). These results are slightly different from those of the previous studies [5,12]. The reason is that the feature extraction methods used in these studies are different. In previous studies, the HOG or weighted HOG image feature extraction methods were used. In contrast, we used an up-to-date feature extraction method based on CNN. Given the difference of feature extraction, the linear kernel is more suitable for gender recognition than the RBF kernel using CNN-based features. As shown in the previous method by Guo et al. [11], which used the BIFs feature extraction method for image feature extraction, the linear kernel is also the best kernel compared to other types of SVM kernels. This result is attributed to the similarity of the BIFs feature extraction method to the CNN-based method. The difference between the two methods is that the BIFs method uses predesigned Gabor kernels as filters for the convolution layers, whereas the CNN-based method trains the filters according to input data and the corresponding ground-truth labels. Consequently, the performance of the CNN-based feature is better than the BIFs features. In addition, although the image features are extracted by a trained CNN-based model, the redundant information can be associated with these features because of the noise in the input images, the characteristics of the training data, and the large variation in the human body images (such as the difference in clothes, hair styles, accessories, and other factors). Consequently, the use of PCA can enhance the recognition accuracy of the system.

## 4. Conclusions

As a new method for gender recognition, we designed the CNN model with respect to the gender information, and used the trained CNN model as the feature extractor. By combining the extracted CNN features from two different types of human body images (visible-light and thermal images), our proposed method can recognize the gender with an error (EER) of 11.439%, which is lower than that of previous methods. In detail, by comparing the error by the conventional structure of CNN, we found that the errors by this structure were 17.216% and 16.610% with visible-light and thermal images, respectively, which were higher than that by the proposed method (11.439%). In addition, by comparing the errors only by visible-light images, thermal images, and both images based on our method, we found that the error by both images was 11.439%, which was much lower than that by visible-light images (17.064%) and by thermal images (16.114%). For the combination of visible-light and thermal images, the recognition errors based on feature-level and score-level fusions were compared. As a result, we found that the error by feature-level fusion was 11.439%, which was lower than that by score-level fusion (11.713%).

Through the last comparison, we found that the proposed method outperforms the previous studies using the same database. In detail, we obtained an error (EER) of 11.439%, much lower than previous results using the HOG method (16.277%), EWHOG method (14.135%), and wHOG method (13.060%). These results confirm that the combination of visible-light and thermal images of the human body and the CNN method for image feature extraction outperforms the previous studies on the body-based gender recognition problem in surveillance systems.

## Figures and Tables

**Table 1:** Summary of previous studies on body-image-based gender recognition.

Categories	Methods	Strength	Weakness
Using a pre-designed (hand-designed) feature extractor for extracting image features.	- Using gait or 3D shape information [13,14,15,16].	- High recognition accuracy can be obtained.	- Requires a series of human body images. - Requires the cooperation of users in the image acquisition step. - Uses a predesigned feature extraction method (features). - Requires an expensive capturing device (scanner) to obtain 3D information of the human body [15,16].
	- Using the HOG or BIFs feature extraction method in only single visible-light images [10,11].	- Easy to implement.	- Limits recognition accuracy because of the use of predesigned and weak feature extraction methods (HOG and BIFs features).
	- Using HOG feature in the combined visible-light and thermal images [5].	- Easy to implement. - Enhances recognition accuracy by utilizing both visible-light and thermal images of the human body.	
	- Using a weighted HOG feature in combined visible-light and thermal images [12].	- Compensates the effects of background regions on recognition accuracy by applying weight values on HOG features. - Enhances recognition accuracy by utilizing both visible-light and thermal images of the human body.	- Limits recognition accuracy because of the use of a predesigned and weak feature extraction method (weighted HOG feature).
Using a leaning-based feature extractor method for extracting image features (**proposed method**)	- Learns the feature extractor using CNN for extracting image features.	- Extracts the more suitable image features for recognition using a pre-trained feature extractor model based on CNN. - Higher recognition accuracy can be obtained compared to predesigned feature extractor methods, such as HOG, BIFs, or weighted HOG.	- Needs training time to train the feature extractor (CNN model).

**Table 2:** Detailed structure description of our proposed CNN method for the gender recognition problem.

Layer Name	Number of Filters	Filter Size	Stride Size	Padding Size	Window Channel Size	Dropout Probability	Output Size
Input Layer	n/a	n/a	n/a	n/a	n/a	n/a	183 × 119 × 1
**Convolution Layer 1**	96	11 × 11 × 1	2 × 2	0	n/a	n/a	87 × 55 × 96
Rectified Linear Unit	n/a	n/a	n/a	n/a	n/a	n/a	87 × 55 × 96
Cross-Channel Normalization Layer	n/a	n/a	n/a	n/a	5	n/a	87 × 55 × 96
MAX Pooling Layer 1	1	3 × 3	2 × 2	0	n/a	n/a	43 × 27 × 96
**Convolution Layer 2**	128	5 × 5 × 96	1 × 1	2 × 2	n/a	n/a	43 × 27 × 128
Rectified Linear Unit	n/a	n/a	n/a	n/a	n/a	n/a	43 × 27 × 128
Cross-Channel Normalization Layer	n/a	n/a	n/a	n/a	5	n/a	43 × 27 × 128
MAX Pooling Layer 2	1	3 × 3	2 × 2	0	n/a	n/a	21 × 13 × 128
**Convolution Layer 3**	256	3 × 3 × 128	1 × 1	1 × 1	n/a	n/a	21 × 13 × 256
Rectified Linear Unit	n/a	n/a	n/a	n/a	n/a	n/a	21 × 13 × 256
**Convolution Layer 4**	256	3 × 3 × 256	1 × 1	1 × 1	n/a	n/a	21 × 13 × 256
Rectified Linear Unit	n/a	n/a	n/a	n/a	n/a	n/a	21 × 13 × 256
**Convolution Layer 5**	128	3 × 3 × 256	1 × 1	1 × 1	n/a	n/a	21 × 13 × 128
Rectified Linear Unit	n/a	n/a	n/a	n/a	n/a	n/a	21 × 13 × 128
MAX Pooling Layer 5	1	3 × 3	2 × 2	0	n/a	n/a	10 × 6 × 128
**Fully Connected Layer 1**	n/a	n/a	n/a	n/a	n/a	n/a	4096
Rectified Linear Unit	n/a	n/a	n/a	n/a	n/a	n/a	4096
**Fully Connected Layer 2**	n/a	n/a	n/a	n/a	n/a	n/a	1024
Rectified Linear Unit	n/a	n/a	n/a	n/a	n/a	n/a	1024
Dropout Layer	n/a	n/a	n/a	n/a	n/a	50%	1024
**Output Layer**	n/a	n/a	n/a	n/a	n/a	n/a	2
Softmax Layer	n/a	n/a	n/a	n/a	n/a	n/a	2
Classification Layer	n/a	n/a	n/a	n/a	n/a	n/a	2

**Table 3:** Description of our self-established collected database for our experiments (10 visible-light images/person and 10 corresponding thermal images/person).

Database	Males	Females	Total
Number of persons	254	158	412 (persons)
Number of images	5080	3160	8240 (images)

**Table 4:** Description of the training and testing sub-databases and the corresponding augmented databases for our experiments.

Database			Males	Females	Total
Augmented database	Learning database	Number of persons	204 (persons)	127 (persons)	331 (persons)
		Number of images	73,440 (204 × 20 × 18 images)	76,200 (127 × 20 × 30 images)	149,640 (images)
	Testing database	Number of persons	50 (persons)	31 (persons)	81 (persons)
		Number of images	18,000 (50 × 20 × 18 images)	18,600 (31 × 20 × 30 images)	36,600 (images)

**Table 5:** Recognition accuracy (EER) of a recognition system that uses only visible-light or thermal images for the recognition problem using CNN (unit: %).

Accuracies of Recognition Systems Using Single Image Types					
Using only Visible-Light Images			Using only Thermal Images		
EER	FAR	GAR	EER	FAR	GAR
17.216	10.000	71.589	16.610	10.000	72.099
	15.000	80.299		15.000	81.350
	**17.220**	**82.786**		**16.610**	**83.387**
	20.00	85.327		20.00	87.285
	25.00	88.532		25.00	91.457

**Table 6:** Recognition accuracy (EER) of a recognition system that uses only visible-light or thermal images for the recognition problem using SVM and CNN features without PCA (unit: %).

SVM Kernel	Accuracies of Recognition Systems Using Single Image Types					
	Using only Visible-Light Images			Using only Thermal Images		
	EER	FAR	GAR	EER	FAR	GAR
Linear	17.379	10.000	72.541	16.560	10.000	73.228
		15.000	80.085		15.000	81.392
		**17.380**	**82.622**		**16.580**	**83.460**
		20.00	85.239		20.00	87.361
		25.00	88.618		25.00	91.471
RBF	17.379	10.000	72.565	16.510	10.000	73.082
		15.000	80.058		15.000	81.471
		**17.400**	**82.642**		**16.520**	**83.500**
		20.00	85.055		20.00	87.475
		25.00	88.604		25.00	91.577

**Table 7:** Recognition accuracy (EER) of a recognition system that uses only visible-light or thermal images for the recognition problem using SVM and CNN features with PCA (unit: %).

SVM Kernel	Accuracies of Recognition Systems Using Single Image Types					
	Using only Visible-Light Images			Using only Thermal Images		
	EER	FAR	GAR	EER	FAR	GAR
Linear	17.064	10.000	73.261	16.114	10.000	73.744
		15.000	80.906		15.000	82.518
		**17.080**	**82.952**		**16.120**	**83.892**
		20.000	85.607		20.000	88.079
		25.000	88.583		25.000	91.758
RBF	17.489	10.000	68.958	17.596	10.000	69.433
		15.000	79.090		15.000	78.042
		**17.500**	**82.523**		**17.600**	**82.409**
		20.000	84.627		20.000	85.149
		25.000	87.055		25.000	89.830

**Table 8:** Recognition accuracy (EER) of recognition systems using a combination of visible-light and thermal images for the recognition problem without PCA (unit: %).

First SVM Layer Kernel	Accuracy of Recognition Systems Using Combined Visible-Light and Thermal Images						
	Feature-Level Fusion Approach			Score-Level Fusion Approach			
	EER	FAR	GAR	Second SVM layer kernel	Accuracy		
					EER	FAR	GAR
Linear	11.766	5.000	73.356	Linear	11.919	5.000	73.684
						10.000	85.297
		10.000	85.465			**11.940**	**88.102**
						15.000	91.214
		**11.780**	**88.247**			20.000	94.388
				RBF	11.956	5.000	73.271
		15.000	91.280			10.000	85.339
						**11.960**	**88.048**
		20.000	94.488			15.000	91.169
						20.000	94.618
RBF	11.684	5.000	73.394	Linear	11.850	5.000	74.008
						10.000	85.206
		10.000	85.689			**11.860**	**88.160**
						15.000	91.402
		**11.700**	**88.332**			20.000	94.627
				RBF	11.956	5.000	73.201
		15.000	91.545			10.000	85.292
						**11.960**	**88.049**
		20.000	94.692			15.000	91.288
						20.000	94.670

**Table 9:** Recognition accuracy (EER) of recognition systems using a combination of visible-light and thermal images for the recognition problem with PCA (unit: %).

First SVM Layer Kernel	Accuracy of Recognition Systems Using Combined Visible-Light and Thermal Images						
	Feature-Level Fusion Approach			Score-Level Fusion Approach			
	EER	FAR	GAR	Second SVM Layer Kernel	Accuracy		
					EER	FAR	GAR
Linear	**11.439**	5.000	76.014	Linear	11.849	5.000	74.966
						10.000	85.834
		10.000	86.570			**11.860**	**88.161**
						15.000	91.274
		**11.440**	**88.561**			20.000	94.270
				RBF	11.863	5.000	73.472
		15.000	92.087			10.000	85.875
						**11.880**	**88.155**
		20.000	94.787			15.000	91.286
						20.000	94.490
RBF	12.808	5.000	71.375	Linear	**11.713**	5.000	73.008
						10.000	85.933
		10.000	81.986			**11.720**	**88.294**
						15.000	91.483
		**12.820**	**87.205**			20.000	94.297
				RBF	11.753	5.000	72.040
		15.000	89.346			10.000	85.781
						**11.760**	**88.253**
		20.000	92.534			15.000	91.530
						20.000	94.368

**Table 10:** Summary of the recognition accuracy of our proposed recognition system in comparison with previous studies (unit: %).

Method	Using Single Visible-Light Images	Using Single Thermal Images	Feature-Level Fusion	Score-Level Fusion
HOG+SVM [5]	17.817	20.463	16.632	16.277
EWHOG+SVM [12]	15.113	19.198	14.767	14.135
wHOG+SVM [12]	15.219	18.257	14.819	13.060
**Our method**	17.064	16.114	**11.439**	**11.713**
